# Knockdown effects of pyrethroid- and neonicotinoid-treated netting against the sand fly *Phlebotomus papatasi* (Diptera: Psychodidae)

**DOI:** 10.1186/s13071-026-07412-2

**Published:** 2026-04-25

**Authors:** Victor I. Agbajelola, Walter D. Roachell, Joshua D. Bast, Mauricio E. Solis, Anthony J. Ramutkowski, Tobin E. Rowland, Gillian S. Lane, Ram K. Raghavan

**Affiliations:** 1 Pathobiology and Integrative Biomedical Sciences, College of Veterinary Medicine, University of Missouri, Columbia, Missouri USA; 2 Public Health Command West, Joint Base San Antonio, San Antonio, Texas USA; 3 Entomological Sciences Division, Defense Centers for Public Health - Aberdeen, Defense Health Agency, Aberdeen, Maryland USA; 4 Air Education and Training Command, Joint Base San Antonio, San Antonio, Texas USA; 5 Entomology Branch, Center for Infectious Disease Research, Walter Reed Army Institute of Research, Silver Spring, Maryland USA; 6 Department of Public Health, College of Health Sciences, University of Missouri, Columbia, Missouri USA; 7 MU Institute for Data Science and Informatics, University of Missouri, Columbia, Missouri USA

**Keywords:** Insecticide-treated netting, Knockdown bioassay, Leishmaniasis vector control, Neonicotinoids, Pyrethroids

## Abstract

**Background:**

Phlebotomine sand flies are important vectors of leishmaniasis and pose persistent risks to human and animal health in endemic and operational environments, including military settings. Barrier-based vector control strategies, such as insecticide-treated netting, provide a practical means of reducing sand fly contact. However, comparative data on the performance of different insecticide classes applied to barrier materials remain limited. This study evaluated the knockdown efficacy of two commercially available insecticides, the pyrethroid esfenvalerate and the neonicotinoid dinotefuran, applied to high-blockage (80%) barrier netting against laboratory-reared *Phlebotomus papatasi*.

**Methods:**

Adult sand flies were exposed to treated and untreated netting for contact durations of 3, 6, 9, 12, and 15 min. Knockdown responses were quantified using standardized laboratory bioassays. The effects of insecticide treatment and exposure time and their interaction were assessed using two-way analysis of variance, with model assumptions verified through residual diagnostics and pairwise comparisons. Probit analyses were conducted to estimate exposure–response relationships and median knockdown times.

**Results:**

Knockdown increased significantly with exposure time for both insecticide treatments, whereas no knockdown was observed in untreated controls. Significant effects of treatment (*P* < 0.001) and exposure time (*P* < 0.001) and their interaction (*P* < 0.001) were detected, indicating treatment-dependent temporal differences in knockdown response. Both insecticides produced significantly greater knockdown than controls, with no significant difference in overall mean knockdown between esfenvalerate and dinotefuran (*P* = 0.89). Although temporal differences in knockdown patterns were observed, probit modeling did not detect a statistically significant difference in fitted slopes between treatments.

**Conclusions:**

Both insecticide-treated barrier nets induced rapid, exposure-dependent knockdown in *P. papatasi*. These findings support the use of pyrethroid- and neonicotinoid-treated barrier netting as effective chemical barriers within integrated sand fly control strategies to reduce leishmaniasis risk in military and endemic environments.

**Graphical Abstract:**

**Figure:**
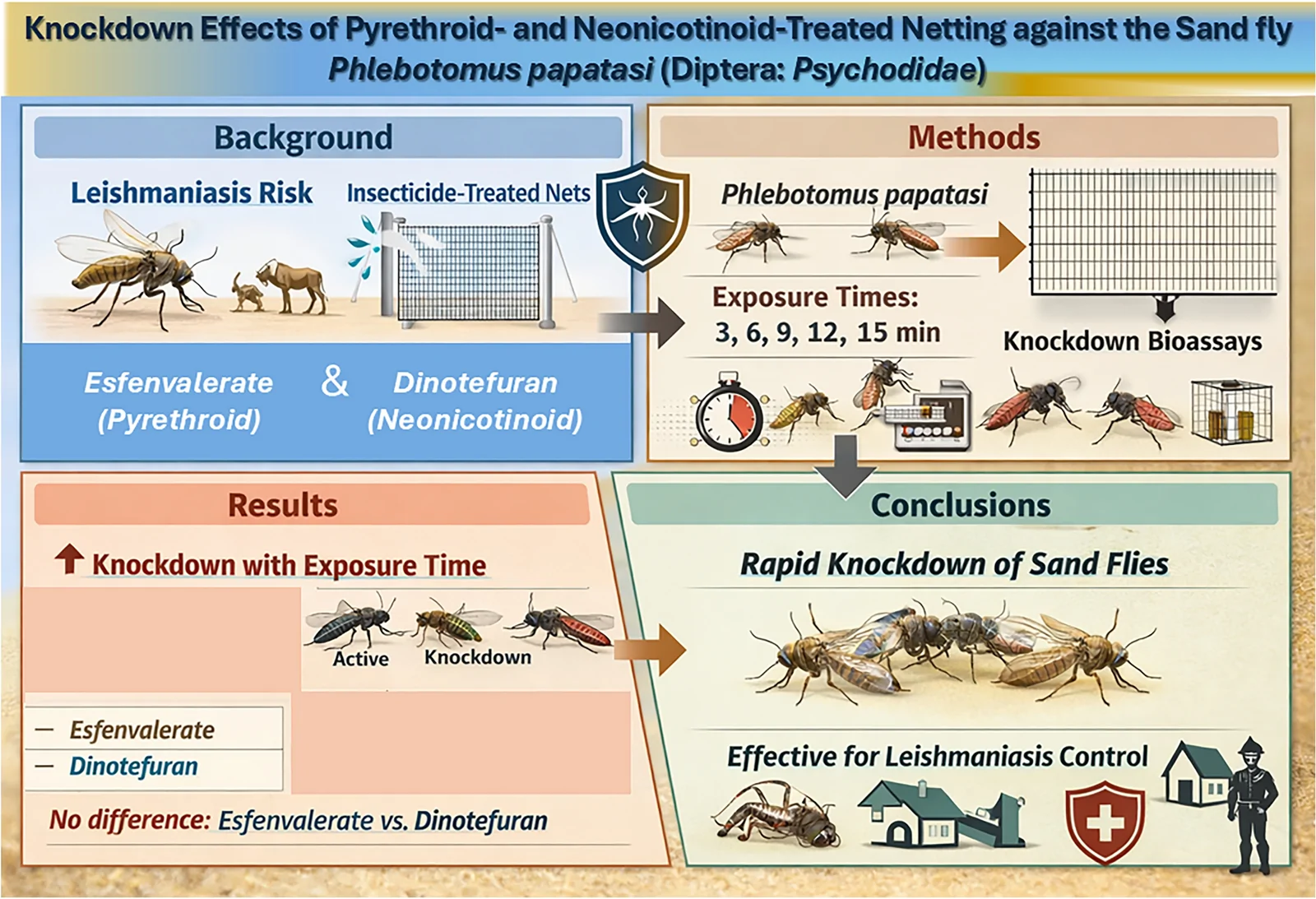

## Background

Sand-fly-borne diseases represent a persistent and expanding global public health challenge, particularly in tropical and subtropical regions where environmental conditions favor vector survival and transmission [1]. Phlebotomine sand flies (Diptera: Psychodidae) are small, nocturnal, hematophagous insects, of which only females transmit pathogens of medical and veterinary importance [1, 2]. Most notably, sand flies serve as biological vectors of *Leishmania* sp., protozoan parasites responsible for leishmaniasis in humans and animals, which manifests in several clinical forms, ranging from self-limiting cutaneous lesions to severe mucocutaneous and visceral disease, and collectively affects millions of people worldwide, with substantial morbidity, social stigma, and economic burden in endemic regions [3]. Among Old World vectors, *Phlebotomus papatasi* is a principal vector of zoonotic cutaneous leishmaniasis (ZCL), transmitting *Leishmania major* across North Africa, the Middle East, and parts of Central and South Asia [4, 5]. Infection typically produces chronic ulcerative skin lesions that may persist for months, often resulting in scarring, secondary bacterial infections, and long-term psychosocial consequences [6].

Beyond endemic civilian populations, sand-fly-borne leishmaniasis poses a well-documented risk to military personnel operating in endemic regions [7, 8]. According to the Centers for Disease Control and Prevention (CDC), during the US military operations in Iraq and Afghanistan, more than 500 parasitologically confirmed cases of cutaneous leishmaniasis were reported among deployed service members, with transmission largely attributed to nighttime exposure in peri-domestic and semi-urban environments where *P. papatasi* is highly active [9]. Subsequent reviews of military deployments have reported cumulative incidence rates approaching 10% in certain operational settings, underscoring the disease’s impact on troop health, force readiness, and mission sustainability [10]. Although *P. papatasi* is not established in the continental USA, phlebotomine sand flies capable of transmitting *Leishmania* spp. are present in several southern states, with highest densities at forest–grassland interfaces and at Fort Campbell, KY, and lower densities in agricultural areas and at Fort Bragg, NC [11, 12], and autochthonous cases of cutaneous leishmaniasis have been documented in Texas and adjacent regions [13, 14]. In parallel, military working dogs (MWDs) are susceptible to leishmaniasis, particularly *Leishmania infantum*, and infected dogs may act as reservoirs under certain ecological conditions, further complicating disease control in military and peri-domestic environments [15]. Together, these factors highlight the relevance of sand fly control not only in overseas deployments but also within domestic and joint human–animal health contexts.

Control of *P. papatasi* and other sand fly vectors relies on an integrated approach informed by vector biology, behavior, and ecology; since sand flies are weak fliers, exhibit crepuscular and nocturnal activity, and possess small body sizes (approximately 2–3 mm), conventional mosquito control tools are often insufficient when used alone [1, 3]. Personal protective measures such as fine-mesh bed nets, topical repellents, and insecticide-treated clothing are therefore central to reducing human–vector contact, particularly in military field settings. However, sand flies can readily pass through standard mosquito netting unless mesh apertures are sufficiently small or nets are supplemented with insecticidal treatments [16, 17]. Chemical interventions, including indoor residual spraying (IRS) with pyrethroids and ultra-low-volume (ULV) space spraying, can achieve high short-term adult mortality, but their effectiveness is often constrained by limited residual activity, operational challenges, and environmental variability [18, 19].

Given the continued public health burden of sand-fly-borne leishmaniasis and its demonstrated impact on military personnel and MWDs, systematic evaluation of vector control tools remains essential, and insecticide-treated barrier netting represents a promising intervention that combines physical exclusion with rapid chemical knockdown, thereby reducing vector contact and potential pathogen transmission. However, comparative data on the knockdown performance of different insecticide classes against *P. papatasi* remain limited, particularly under controlled exposure scenarios relevant to military applications. This study therefore aimed to evaluate and compare the knockdown efficacy of two commercially available insecticides, the pyrethroid–esfenvalerate and the neonicotinoid–dinotefuran, applied to high-blockage (80%) 300 Series netting against *Phlebotomus papatasi*. By quantifying time-dependent knockdown responses and characterizing exposure–response relationships, this work seeks to inform evidence-based vector control strategies for protecting military personnel, military working dogs, and other at-risk populations in endemic and high-exposure settings.

Environmental management strategies, including rodent burrow treatment, habitat modification, and targeting of immature stages to reduce adult emergence, have been implemented to suppress sand fly populations; however, their effectiveness is often limited by the cryptic and poorly characterized nature of sand fly breeding sites [20, 21]. Although repellents containing 10–30% *N*,*N*-diethyl-*meta*-toluamide (DEET) can provide approximately 90% protection against sand fly bites, and permethrin-treated clothing has been shown to reduce biting rates by up to 70%, these measures depend on consistent user compliance and may not fully prevent exposure under high-risk conditions [18, 22]. Moreover, emerging reports of insecticide tolerance and resistance further complicate control efforts, underscoring the need to evaluate alternative insecticide classes, formulations, and delivery systems that may improve efficacy and durability in operational environments [23, 24].

## Methods

Laboratory-reared *Phlebotomus papatasi* (North Sinai strain; late-third- and fourth-instar larvae or pupae) were obtained from the Walter Reed Army Institute of Research (WRAIR) through BEI Resources (NR-43999). Upon arrival, larval rearing containers were inspected for physical integrity and immediately placed in an environmental incubator maintained at 26 °C, 80% relative humidity, and a 12:12 h light:dark photoperiod. Because the shipment consisted exclusively of late larval stages or pupae, supplemental larval feeding was not required. Rearing pots were examined twice weekly to monitor adult emergence, fungal growth, and moisture content of the plaster substrate, and any pots containing emerged adults were opened directly within screened adult holding cages to allow natural dispersal. Pots without evidence of emergence were gently agitated to disrupt mold formation and prevent fungal overgrowth that could impede development, and when plaster substrates appeared excessively dry, rehydration was achieved by placing pots in approximately 2.5 cm of water for 5–10 min to prevent desiccation of immature stages.

Emergent adults were maintained in screened holding cages housed within an incubator set to 25 °C, 80% relative humidity, and a 12:12 h light:dark photoperiod, with continuous access to a carbohydrate source provided via cotton pads saturated with a 30% sucrose solution prepared from commercially available granulated sugar. Cotton pads were lightly compressed to prevent dripping and replaced daily to maintain consistent nutrition and hygienic conditions. On each experimental day, adult sand flies that were 3–5 days post-emergence were transferred to the test room in labeled containers and allowed to acclimatize for a minimum of 1 h prior to bioassay initiation, a step implemented to minimize potential effects of environmental transition and handling stress on knockdown responses. Transfers were performed using a manual aspirator fitted with a modified serological pipette, created by shortening and trimming a 10 mL pipette to produce an aperture suitable for sand fly aspiration and securing it to the aspirator with adhesive tape, thereby facilitating controlled transfer while minimizing injury or stress to the insects.

High-density barrier netting (300 Series, 80% blockage camouflage netting; FenceScreen, LLC, Oxford, NC, USA) was selected on the basis of its relevance to protective applications in US military environments, particularly facilities housing military working dogs (MWDs). Netting was treated on 13 May 2025 at the MWD kennel complex located at Fort Sam Houston, Texas, USA, to reflect realistic operational deployment conditions. Two commercially available insecticide formulations representing distinct chemical classes were evaluated, including dinotefuran, a neonicotinoid insecticide (Alpine^®^ WSG: EPA Reg No 499-561), applied as a 0.3% solution (30 g per gallon of water), and esfenvalerate, a pyrethroid insecticide (Onslaught^®^ FastCap: EPA Reg No 1021-1815), applied as a 0.5% solution (1 oz per gallon of water).

Insecticides were prepared and applied according to manufacturer label instructions using a calibrated hand-held compression sprayer to ensure uniform coverage, with netting panels suspended vertically along the perimeter fencing of the kennel facility to simulate operational deployment as barrier materials. Following application, nets were allowed to air-dry under ambient outdoor conditions for 24 h. After this drying period, large net sections were collected from multiple locations across each treated and untreated panel to capture potential spatial variability in insecticide deposition and transported to the University of Missouri for bioassay testing. Upon receipt the following day, these sections were further subdivided into standardized clippings (8.2 × 8.2 cm) using sterilized scissors, individually labeled, and maintained under room temperature and light-protected conditions until use.

Clippings were obtained from randomized positions across each net panel to ensure a representative distribution of insecticide exposure. For each treatment–exposure-time combination, a single clipping was used consistently across all replicates to maintain uniformity of the treated surface, while replication was achieved through repeated bioassays using new groups of sand flies rather than different net samples. The short interval between treatment, transport, and testing minimized potential degradation, ensuring that insecticidal activity was retained at the time of bioassay.

The study followed a completely randomized design consisting of three treatment groups (esfenvalerate-treated, dinotefuran-treated, and untreated-control netting), each evaluated across five exposure durations (3, 6, 9, 12, and 15 min), with five independent replicates per treatment–time combination, with each replicate consisting of 5 adult sand flies, yielding 25 insects per exposure duration and 125 insects per treatment group.

Knockdown bioassays were conducted using a contact-exposure system adapted from the standard World Health Organization (WHO) cone bioassay setup and modified to accommodate the small size and behavioral characteristics of sand flies. Individual exposure cones (3.5 inches in diameter and approximately 3 inches in height) were fully lined internally with treated net clippings and positioned at an approximate 45° incline to promote active contact with the surface of the treated net clippings. Adult sand flies were introduced using a manual aspirator and confined for the assigned exposure duration, after which they were gently transferred into labeled observation containers supplied with a 30% sucrose source and maintained under standard insectary conditions. The exposure design ensured continuous and repeated contact between sand flies and the treated material while allowing sufficient space for movement within the cones. Knockdown was assessed at 1 h post-exposure and defined as the inability of an insect to stand upright or exhibit coordinated movement following gentle stimulation. Mortality was also recorded at 24 h post-exposure to capture potential delayed effects; however, these data were not included in the present analysis, as the study was specifically designed to evaluate immediate knockdown as an indicator of rapid insecticidal action under short exposure durations.

Experimental observations were recorded on standardized data sheets and subsequently entered into Microsoft Excel^®^ with independent verification to minimize transcription errors. Recorded variables included treatment group, exposure duration, replicate number, number of insects exposed, and number knocked down at 1 h post-exposure, and knockdown responses were expressed as percentages relative to the number exposed for each treatment and exposure duration. All statistical analyses were conducted using R software (version 4.5.1) [25], with descriptive summaries generated using the *dplyr* package and graphical visualizations produced with *ggplot2* [26]. The effects of insecticide treatment and exposure time and their interaction on knockdown response were evaluated using a two-way analysis of variance (ANOVA), with model assumptions assessed through inspection of residual diagnostics and pairwise comparisons among treatment groups performed using Tukey’s honestly significant difference (HSD) test at a family-wise confidence level of 95%.

To further characterize exposure–response relationships, knockdown data were analyzed using treatment-specific generalized linear models with a binomial error distribution and probit link function, incorporating log_10_-transformed exposure time as the predictor. Model fit was evaluated using Pearson residuals, and dispersion parameters were calculated to assess potential overdispersion. Probit models were used to estimate median knockdown times (KT_50_) and high-level knockdown times (KT_90_), with corresponding 95% confidence intervals (CIs) derived using the delta method, and nested probit models assuming common and treatment-specific slopes were compared using likelihood ratio tests based on Chi square statistics to determine whether exposure–response slopes differed among insecticide treatments. All statistical tests were two-sided, statistical significance was defined at *α* = 0.05, and results are presented as model estimates with associated standard errors (SEs), confidence intervals, and *P*-values, as appropriate.

## Results

**Fig. 1:**
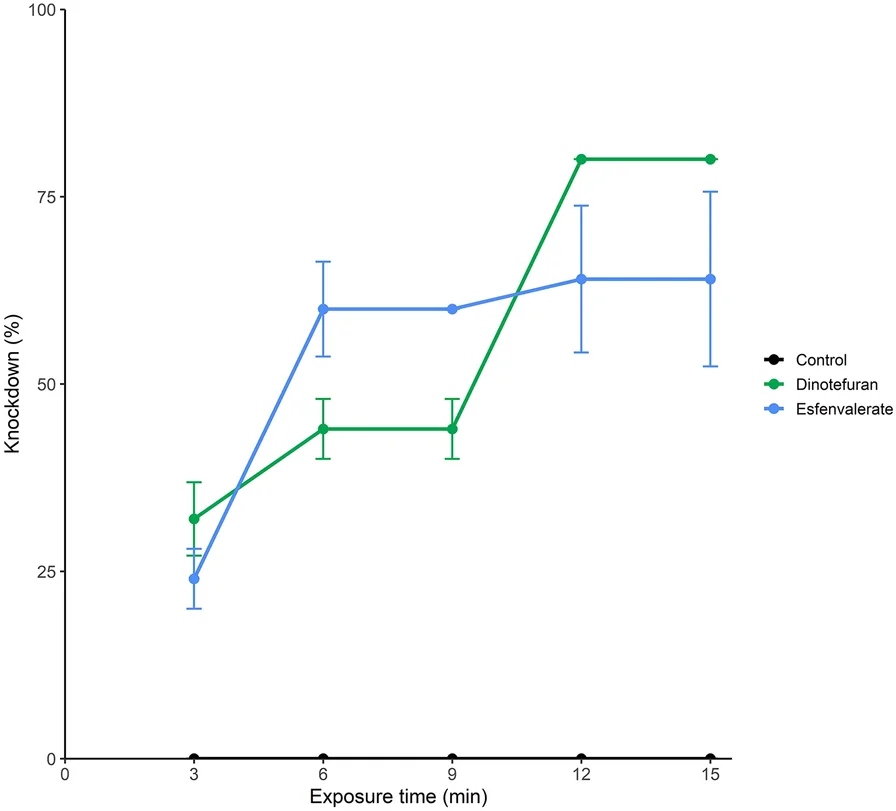
Knockdown effect of insecticide-treated netting over exposure time. Mean knockdown percentages (± standard error) of *Phlebotomus papatasi* following exposure to 300 Series 80% blockage netting treated with esfenvalerate (Onslaught^®^) or dinotefuran (Alpine^®^) across five exposure durations (3–15 min). Control netting produced no knockdown at any time point. Points represent mean knockdown values, and error bars indicate standard errors across replicates

Knockdown of *Phlebotomus papatasi* increased with exposure time for both insecticide-treated nets, while no knockdown was observed in the untreated control at any exposure duration (Fig. 1). Esfenvalerate-treated netting (Onslaught^®^) induced a rapid initial response, achieving approximately 60% knockdown within 6 min, remaining at similar levels through 15 min. In contrast, dinotefuran-treated netting (Alpine^®^) showed a delayed onset followed by a marked increase in knockdown between 9 and 12 min, ultimately reaching levels comparable to or exceeding those observed for esfenvalerate at longer exposure times. Variability in knockdown responses increased at extended exposure durations, particularly for esfenvalerate-treated netting, as indicated by wider confidence intervals.

Two-way analysis of variance revealed significant main effects of insecticide treatment (*F*_2,69_ = 165.77, *P* < 0.001) and exposure time (*F*_1,69_ = 50.73, *P* < 0.001) on knockdown, as well as a significant treatment × time interaction (*F*_2,69_ = 14.56, *P* < 0.001), indicating that knockdown dynamics differed between insecticides across exposure durations. Tukey’s HSD post hoc comparisons showed that both dinotefuran- and esfenvalerate-treated nets produced significantly higher knockdown than the untreated control, with mean differences of 56.0% (95% CI 47.6–64.4, *P* < 0.001) and 54.4% (95% CI 46.0–62.8, *P* < 0.001). No statistically significant difference in voerall knockdown was observed between esfenvalerate and dinotefuran (mean difference =  −1.6%, 95% CI −10.0 to 6.8, *P* = 0.89), indicating comparable average performance across exposure durations despite temporal differences in knockdown patterns (Table 1).

**Table 1:** Pairwise comparisons of knockdown percentages among treatments using Tukey’s HSD test

Comparison	Mean difference (%)	95% CI (lower–upper)	*P*-value
Dinotefuran versus control	56.0	47.6–64.4	< 0.001
Esfenvalerate versus control	54.4	46.0–62.8	< 0.001
Esfenvalerate versus dinotefuran	−1.6	−10.0 to 6.8	0.89

**Fig. 2:**
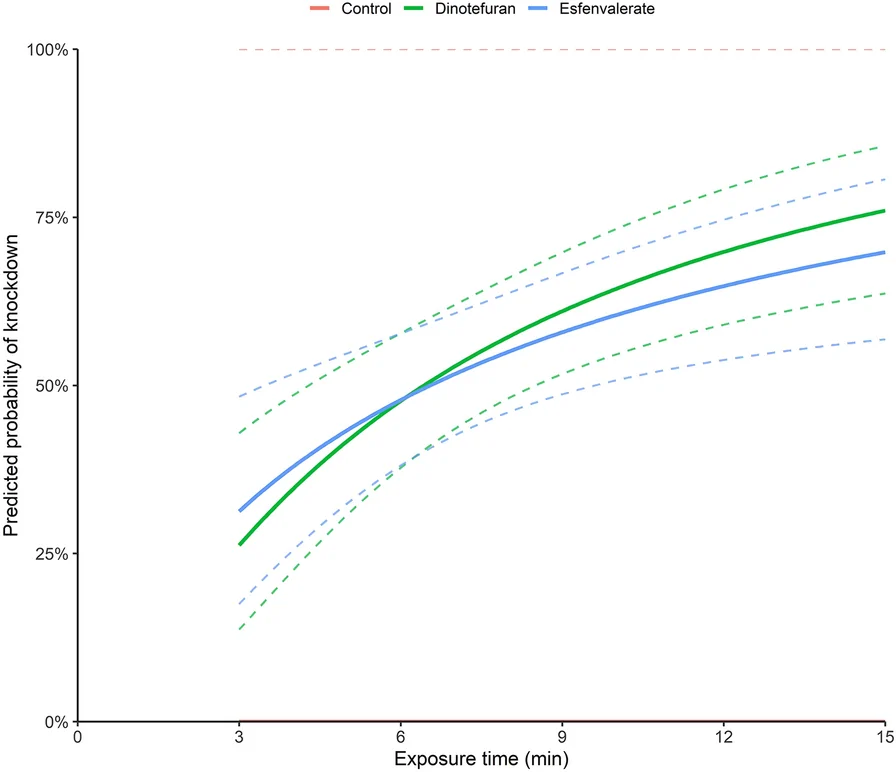
Probit-predicted probability of knockdown of *Phlebotomus papatasi* as a function of exposure time for esfenvalerate-treated, dinotefuran-treated, and untreated netting. Dashed lines indicate 95% confidence intervals around model predictions

Probit regression models were used to further characterize the relationship between exposure duration and knockdown probability (Fig. 2). For both insecticide-treated nets, predicted knockdown probability increased with exposure time, whereas the untreated control remained near zero across the exposure range. Although observed knockdown patterns suggested temporal differences in relative treatment performance, particularly at shorter versus longer exposure durations, likelihood ratio testing indicated that allowing treatment-specific slopes did not significantly improve model fit (*χ*^2^ = 0.50, degrees of freedom [df] = 2, *P* = 0.78). Thus, while the ANOVA detected a significant treatment × exposure time interaction on the observed knockdown scale, the probit analysis on the log-transformed exposure scale did not support a statistically significant difference in fitted slopes between insecticides.

Treatment-specific probit models confirmed a significant positive association between exposure time and knockdown probability for both insecticides. For esfenvalerate-treated netting, the slope coefficient for log_10_-transformed exposure time was positive and significant (*β* = 1.44 ± 0.47 SE, *P* = 0.002). Dinotefuran-treated netting showed a similarly significant but steeper exposure–response relationship (*β* = 1.92 ± 0.49 SE, *P* < 0.001) (Table 2). Intercept estimates differed between treatments, with the more negative intercept for dinotefuran indicating lower initial knockdown at minimal exposure followed by a sharper increase as contact time increased. Probit models for the untreated control showed no meaningful association between exposure time and knockdown, with non-significant slope and intercept estimates consistent with the absence of insecticidal activity.

**Table 2:** Probit regression coefficients describing the relationship between exposure time and knockdown response

Treatment	Term	Estimate (*β*)	SE	*z*-Value	*P*-value	Dispersion
Esfenvalerate	Intercept	−1.18	0.44	−2.69	0.007	0.68
	Log_10_ (exposure time)	1.44	0.47	3.05	0.002	
Dinotefuran	Intercept	−1.55	0.45	−3.45	< 0.001	0.33
	Log_10_ (exposure time)	1.92	0.49	3.94	< 0.001	
Control	Intercept	−6.97	23 357	−0.0003	1.00	~0
	Log_10_ (exposure time)	~0	25 211	~0	1.00	

Probit-derived knockdown time estimates indicated rapid effects for both insecticide-treated nets, with similar median knockdown times. The KT_50_ for esfenvalerate-treated netting was estimated at 6.55 (95% CI 4.49–9.55), while dinotefuran-treated netting produced a comparable KT_50_ of 6.43 (95% CI 4.81–8.61). At higher knockdown thresholds, dinotefuran-treated netting achieved 90% knockdown more rapidly (KT_90_ = 29.9; 95% CI 14.5–61.7) than esfenvalerate-treated netting (KT_90_ = 50.8; 95% CI 14.4–178), although confidence intervals overlapped, indicating uncertainty at higher exposure levels. KT values could not be calculated for the control group due to the absence of measurable knockdown, resulting in non-finite estimates (Table 3).

**Table 3:** Probit-derived KT_50_ and KT_90_ estimates for sand flies (*P. papatasi*) exposed to treated and untreated netting

Treatment	Metric	Estimate (min)	95% CI (min)
Esfenvalerate	KT_50_	6.55	4.49–9.55
	KT_90_	50.8	14.4–178
Dinotefuran	KT_50_	6.43	4.81–8.61
	KT_90_	29.9	14.5–61.7
Control	KT_50_	–	–
	KT_90_	—	—

Dispersion parameters for the probit models were below unity for esfenvalerate-treated (0.68) and dinotefuran-treated (0.33) nets, indicating no evidence of overdispersion and supporting the adequacy of the binomial modeling framework. The low dispersion estimates for the control group reflects the near-complete absence of knockdown events rather than true model precision. Comparison of models with common versus treatment-specific slopes showed no significant difference in fit (*χ*^2^ = 0.50, df = 2, *P* = 0.78), indicating that the rate of increase in knockdown with exposure time did not differ significantly between insecticides.

## Discussion

This study quantified the knockdown responses of *P. papatasi* to two commercially available insecticides—esfenvalerate (pyrethroid) and dinotefuran (neonicotinoid)—applied to high-blockage netting under simulated operational conditions. By integrating time-resolved knockdown observations with inferential (ANOVA) and dose–response (probit) modeling, we generated comparative performance metrics (KT_50_, KT_90_) that inform both mechanistic understanding and applied vector control. Across all exposure intervals, knockdown increased with contact duration for both insecticides, while untreated controls showed no response, confirming that observed effects were attributable to insecticidal activity rather than physical interaction with the netting alone. These findings align with prior laboratory studies demonstrating that insecticide-impregnated barriers disrupt sand fly locomotion and host-seeking behavior, even when complete exclusion is not achieved [27, 28].

Although both insecticides produced increasing knockdown with exposure time, distinct temporal differences in response profiles were evident, reflecting treatment-dependent variation in knockdown dynamics. Esfenvalerate produced a more rapid initial knockdown, whereas dinotefuran exhibited a slower onset followed by greater increases at longer exposure durations. These patterns are consistent with differences in mode of action between insecticide classes: pyrethroids act primarily on voltage-gated sodium channels, inducing rapid neuronal excitation, while neonicotinoids target nicotinic acetylcholine receptors, often producing delayed but cumulative neurotoxic effects [29, 30]. Additional factors, including differences in cuticular penetration, formulation bioavailability, and insect contact behavior on treated surfaces, may further contribute to these temporal patterns [22, 31]. Similar class-dependent differences in knockdown timing have been reported in other sand fly species, including *Phlebotomus sergenti* and *Phlebotomus duboscqi*, where insecticide type influenced the rate, but not necessarily the overall magnitude, of response under controlled exposure conditions [32, 33].

The significant effects of treatment and exposure duration and their interaction observed in this study are consistent with prior evaluations of insecticide-treated materials against sand flies and other hematophagous insects. Studies assessing pyrethroid-impregnated nets and curtains against *Phlebotomus argentipes* and *P. sergenti* similarly reported strong time-dependent knockdown with significant treatment × exposure interactions, reflecting compound-specific kinetics rather than categorical differences in susceptibility [34, 35]. In the present study, post hoc analyses indicated that both esfenvalerate and dinotefuran significantly outperformed untreated controls but did not differ significantly from each other overall, suggesting broadly comparable effectiveness when applied to high-blockage netting. This finding is consistent with baseline susceptibility assessments showing that *P. papatasi* remains responsive to multiple insecticide classes under laboratory conditions [36, 37].

Importantly, although both insecticides were effective, neither achieved complete knockdown at early exposure durations, indicating that barrier efficacy depends not only on toxic potency but also on sufficient contact time. Similar patterns have been reported in laboratory and semi-field studies in which sand flies required extended exposure to insecticide-treated surfaces to achieve high levels of knockdown or mortality, particularly under brief or low-dose exposure conditions [32, 33]. These findings highlight the importance of behavioral and environmental factors, including net placement, vector activity patterns, and duration of contact, in determining operational effectiveness.

Knockdown time (KT) estimates further support the practical relevance of both insecticides, with KT_50_ values of approximately 6–7 min indicating rapid impairment under controlled conditions. Comparable KT_50_ estimates have been reported for pyrethroid-treated materials tested against *P. argentipes* and *Lutzomyia longipalpis*, although absolute values vary with species, formulation, and exposure method [34, 38]. The higher KT_90_ observed for esfenvalerate relative to dinotefuran suggests that prolonged exposure may be required to achieve near-complete knockdown with certain pyrethroid formulations, a pattern also observed in other vector control studies evaluating knockdown and mortality kinetics across insecticide classes [33]. Field-based studies using insecticide-treated materials have similarly demonstrated reductions in sand fly biting and landing rates, although responses vary across species and ecological contexts, emphasizing the need for species-specific and context-dependent evaluation [22, 39]. For example, in the Neotropical vector *Lutzomyia longiflocosa*, treated materials produced measurable knockdown and mortality over extended exposure periods, albeit at lower levels than those observed under controlled laboratory conditions in the present study [40].

Although the ANOVA indicated a significant treatment × exposure interaction on observed knockdown percentages, probit modeling based on log-transformed exposure time did not detect a statistically significant difference in fitted slopes between insecticides. This suggests that, while temporal differences in knockdown dynamics were evident, these differences were not sufficient to produce distinct exposure–response gradients under the probit modeling framework. Differences between observed patterns and model-based interpretations may reflect the sensitivity of statistical approaches to scale and transformation, as well as the relatively narrow range of exposure durations evaluated.

Differences between our findings and some prior reports may also reflect the influence of insecticide resistance mechanisms. Pyrethroid resistance in *P. papatasi* has been documented in parts of the Old World and can arise through metabolic detoxification or target-site insensitivity, potentially altering knockdown dynamics over time [41]. Although the present study used laboratory-reared flies with controlled susceptibility, continued surveillance of resistance phenotypes in field populations will be essential for translating these results into durable vector control strategies. Taken together, the demonstrated susceptibility of *P. papatasi* to both pyrethroid and neonicotinoid treatments supports the continued use of insecticide-treated barrier materials in high-risk operational environments, including military deployments.

Sand fly exposure remains a well-documented risk factor for cutaneous leishmaniasis among deployed personnel in endemic regions of Southwest and Central Asia, and recent syntheses of military health data report substantial disease burden despite existing preventive measures, underscoring the need for effective, evidence-based vector control tools as part of integrated force health protection strategies [10]. Within this context, the present study provides quantitative evidence supporting the operational relevance of treated netting and highlights the value of evaluating alternative insecticide classes to enhance resilience against resistance and variability in field performance. These findings support the use of treated netting as a practical intervention for reducing sand fly exposure in high-risk environments, including military operations.

Several limitations should be considered when interpreting these findings. The experiments were conducted under controlled laboratory conditions using standardized bioassays, which ensured reproducibility but did not capture the environmental complexity of operational settings where factors such as temperature fluctuations, humidity, wind, dust accumulation, and host activity may influence sand fly contact behavior and insecticide performance. The use of laboratory-reared *P. papatasi* further constrains extrapolation to field populations, which may display variable susceptibility due to prior insecticide exposure, ecological selection pressures, and emerging resistance mechanisms. The study emphasized short-term knockdown responses and did not assess longer-term mortality, sublethal effects, or behavioral avoidance, all of which are relevant to vector–host interactions and transmission risk. In addition, evaluation was limited to two insecticide classes applied to a single netting configuration, restricting generalization to other formulations, materials, or deployment strategies.

## Conclusions

This study demonstrated that high-blockage netting treated with either esfenvalerate or dinotefuran produces significant, exposure-dependent knockdown in *P. papatasi*, with comparable median knockdown times and statistically similar exposure–response slopes. The absence of knockdown in untreated netting highlights the critical role of insecticidal treatment in disrupting sand fly movement and contact with barrier materials. Together, these findings meet the study objective of quantitatively evaluating the knockdown efficacy of pyrethroid- and neonicotinoid-treated netting and establish robust performance benchmarks relevant to barrier-based sand fly control.

The comparable efficacy observed between the two insecticide classes supports their utility as viable chemical options for treated barrier applications, particularly in operational environments where sand fly exposure presents a documented risk to human and animal health. These results are directly applicable to military and peri-domestic settings, including facilities housing military working dogs, and reinforce the value of insecticide-treated netting as a practical component of force health protection and leishmaniasis risk reduction.

Building on these findings, future work should explore the performance of insecticide-treated netting across a wider range of deployment scenarios, insecticide formulations, and sand fly taxa to strengthen generalizability and inform optimization of control strategies. Longitudinal assessments of residual activity and durability under routine environmental exposure would further inform expectations for sustained operational performance. Given the global concern surrounding insecticide resistance, incorporation of resistance surveillance and susceptibility monitoring into future evaluations will be important for preserving long-term efficacy. Finally, insecticide-treated barrier netting should be integrated within broader vector management frameworks that include environmental management, reservoir control, and personal protective measures to maximize protective impact and sustainability.

## Data Availability

Data supporting the main conclusions of this study are included in the manuscript.

## References

[1] Cecílio P, Cordeiro-da-Silva A, Oliveira F. Sand flies: basic information on the vectors of leishmaniasis and their interactions with *Leishmania* parasites. Commun Biol. 2022;5:305. 10.1038/s42003-022-03240-z. 35379881 PMC8979968

[2] Akhoundi M, Kuhls K, Cannet A, Votýpka J, Marty P, Delaunay P, et al. A historical overview of the classification, evolution, and dispersion of *Leishmania* parasites and sandflies. PLoS Negl Trop Dis. 2016;10:e0004349. 10.1371/journal.pntd.0004349. 26937644 PMC4777430

[3] Courtenay O, Peters NC, Rogers ME, Bern C. Combining epidemiology with basic biology of sand flies, parasites, and hosts to inform leishmaniasis transmission dynamics and control. PLoS Pathog. 2017;13:e1006571. 10.1371/journal.ppat.1006571. 29049371 PMC5648254

[4] Service MW. Phlebotomine sand flies (*Phlebotominae*). In: Medical entomology for students. Cambridge: Cambridge University Press; 2012. p. 98–107.

[5] Pigott DM, Bhatt S, Golding N, Duda KA, Battle KE, Brady OJ, et al. Global distribution maps of the leishmaniases. Elife. 2014;3:e02851. 10.7554/eLife.02851. 24972829 PMC4103681

[6] Munstermann LE. Phlebotomine sand flies and moth flies (*Psychodidae*). In: Mullen GR, Durden LA, editors. Medical and veterinary entomology. 3rd ed. London: Academic Press; 2019. p. 191–211.

[7] Kniha E, Obwaller AG, Dobler G, Poeppl W, Mooseder G, Walochnik J. *Phlebovirus* seroprevalence in Austrian Army personnel returning from missions abroad. Parasit Vectors. 2019;12:416. 10.1186/s13071-019-3674-6. 31445517 PMC6708154

[8] Shiraly R, Khosravi A, Farahangiz S. Seroprevalence of sand fly fever virus infection in military personnel on the western border of Iran. J Infect Public Health. 2017;10:59–63. 10.1016/j.jiph.2016.02.014. 27017407

[9] Centers for Disease Control and Prevention (CDC). Cutaneous leishmaniasis in U.S. military personnel—Southwest/Central Asia, 2002–2003. MMWR Morb Mortal Wkly Rep. 2003;52:1013–6. 14574274

[10] Rawlings N, Bailey M, Courtenay O. Leishmaniasis in deployed military populations: a systematic review and meta-analysis. PLoS Negl Trop Dis. 2025;19:e0012680. 10.1371/journal.pntd.0012680. 40063644 PMC11913291

[11] Claborn D, Masuoka P, Morrow M, Keep L. Habitat analysis of North American sand flies near veterans returning from Leishmania-endemic war zones. Int J Health Geogr. 2008;7:65. 10.1186/1476-072X-7-65. 19094218 PMC2640372

[12] Claborn DM, Rowton ED, Lawyer PG, Brown GC, Keep LW. Species diversity and relative abundance of phlebotomine sand flies (*Diptera*: *Psychodidae*) on three Army installations in the southern USA and susceptibility of a domestic sand fly to infection with Old World *Leishmania major*. Mil Med. 2009;174:1203–8. 10.7205/milmed-d-00-4309. 19960830

[13] Wright NA, Davis LE, Aftergut KS, Parrish CA, Cockerell CJ. Cutaneous leishmaniasis in Texas: a northern spread of endemic areas. J Am Acad Dermatol. 2008;58:650–2. 10.1016/j.jaad.2007.11.008. 18249464

[14] Yao C, Yang Y, Du A. Autochthonous leishmaniasis in the United States of America. Microorganisms. 2025;13:2485. 10.3390/microorganisms13112485. 41304171 PMC12654226

[15] Schaut RG, Robles-Murguia M, Juelsgaard R, Esch KJ, Bartholomay LC, Ramalho-Ortigao M, et al. Vectorborne transmission of *Leishmania infantum* from hounds, United States. Emerg Infect Dis. 2015;21:2209–12. 10.3201/eid2112.141167. 26583260 PMC4672406

[16] Dokhan MR, Kenawy MA, Shaibi T, Annajar BB. Field evaluation of outdoor ultra-low volume (ULV) applications against phlebotomine sand flies (*Diptera*: *Psychodidae*) in Al Rabta, North-West of Libya. J Arthropod Borne Dis. 2017;11:393–402. 29322056 PMC5758635

[17] Balaska S, Fotakis EA, Chaskopoulou A, Vontas J. Chemical control and insecticide resistance status of sand fly vectors worldwide. PLoS Negl Trop Dis. 2021;15:e0009586. 10.1371/journal.pntd.0009586. 34383751 PMC8360369

[18] Khoobdel M. Evaluation of permethrin-treated clothing for personal protection against *Phlebotomus papatasi* (*Diptera*: *Psychodidae*). J Entomol. 2008;5:51–5.

[19] Montenegro-Quiñonez CA, Buhler C, Horstick O, Runge-Ranzinger S, Rahman KM. Efficacy and community-effectiveness of insecticide-treated nets for the control of visceral leishmaniasis: a systematic review. PLoS Negl Trop Dis. 2022;16:e0010196. 10.1371/journal.pntd.0010196. 35235556 PMC8890655

[20] Moncaz A, Faiman R, Kirstein O, Warburg A. Breeding sites of *Phlebotomus sergenti*, the sand fly vector of cutaneous leishmaniasis in the Judean Desert. PLoS Negl Trop Dis. 2012;6:e1725. 10.1371/journal.pntd.0001725. 22802981 PMC3389037

[21] Tsurim I, Wasserberg G, Ben Natan G, Abramsky Z. Systemic control of cutaneous leishmaniasis sand-fly vectors: fipronil-treated rodent bait is effective in reducing *Phlebotomus papatasi* (Diptera: *Psychodidae*) female emergence rate from rodent burrows. J Med Entomol. 2021;58:974–8. 10.1093/jme/tjaa201. 33155657

[22] Maroli M, Feliciangeli MD, Bichaud L, Charrel RN, Gradoni L. Phlebotomine sandflies and the spreading of leishmaniases and other diseases of public health concern. Med Vet Entomol. 2013;27:123–47. 10.1111/j.1365-2915.2012.01034.x. 22924419

[23] Shirani-Bidabadi L, Oshaghi MA, Enayati AA, Akhavan AA, Zahraei-Ramazani AR, Yaghoobi-Ershadi MR, et al. Molecular and biochemical detection of insecticide resistance in the *Leishmania* vector, *Phlebotomus papatasi* (Diptera: *Psychodidae*), to dichlorodiphenyltrichloroethane and pyrethroids, in Central Iran. J Med Entomol. 2022;59:1347–54. 10.1093/jme/tjac031. 35595289

[24] Bezerra-Santos MA, Dantas-Torres F, Maia C, et al. Bio-ecology and management of phlebotomine sand flies: unraveling the complexity of vector control. J Pest Sci. 2026;99:11. 10.1007/s10340-025-01992-1.

[25] R Core Team. R: A language and environment for statistical computing. Vienna: R Foundation for Statistical Computing. 2025. https://www.R-project.org/.

[26] Wickham H, François R, Henry L, Müller K, Vaughan D. dplyr: a grammar of data manipulation. 2023. https://github.com/tidyverse/dplyr.

[27] Rowland T, Davidson SA, Kobylinski K, Menses C, Rowton E. Efficacy of permethrin treated bed nets against *Leishmania major*-infected sand flies. US Army Med Dep J. 2015;10–15. 26276941

[28] Silva JJ, Scott JG. Conservation of the voltage-sensitive sodium channel protein within the *Insecta*. Insect Mol Biol. 2020;29:9–18. 10.1111/imb.12605. 31206812

[29] Tomizawa M, Casida JE. Neonicotinoid insecticide toxicology: mechanisms of selective action. Annu Rev Pharmacol Toxicol. 2005;45:247–68. 10.1146/annurev.pharmtox.45.120403.095930. 15822177

[30] Ffrench-Constant RH, Williamson MS, Davies TG, Bass C. Ion channels as insecticide targets. J Neurogenet. 2016;30:163–77. 10.1080/01677063.2016.1229781. 27802784 PMC6021766

[31] Siddiqui JA, Fan R, Naz H, Bamisile BS, Hafeez M, Ghani MI, et al. Insights into insecticide-resistance mechanisms in invasive species: challenges and control strategies. Front Physiol. 2023;13:1112278. 10.3389/fphys.2022.1112278. 36699674 PMC9868318

[32] Faraj C, Ouahabi S, Adlaoui EB, El Elkohli M, Lakraa L, El Rhazi M, et al. Insecticide susceptibility status of *Phlebotomus (Paraphlebotomus) sergenti* and *Phlebotomus (Phlebotomus) papatasi* in endemic foci of cutaneous leishmaniasis in Morocco. Parasit Vectors. 2012;5:51. 10.1186/1756-3305-5-51. 22429776 PMC3359231

[33] Li AY, Perez de Leon AA, Linthicum KJ, Britch SC, Bast JD, Debboun M. Baseline susceptibility to pyrethroid and organophosphate insecticides in two Old World sand fly species (Diptera: *Psychodidae*). US Army Med Dep J. 2015;3–9. 26276940

[34] Dinesh DS, Das ML, Picado A, Roy L, Rijal S, Singh SP, et al. Insecticide susceptibility of *Phlebotomus argentipes* in visceral leishmaniasis endemic districts in India and Nepal. PLoS Negl Trop Dis. 2010;4:e859. 10.1371/journal.pntd.0000859. 21049013 PMC2964302

[35] Silva GBS, Lopes JV, Pessoa GCA, Pinheiro LC, Dos Santos JP, Lara-Silva FO, et al. Susceptibility of *Lutzomyia longipalpis* (Lutz & Neiva, 1912) to Fludora FusionPM, a combination of clothianidin and deltamethrin: field and laboratory bioassays. Parasit Vectors. 2025;19:49. 10.1186/s13071-025-07206-y. 41437384 PMC12849672

[36] Tetreault GE, Zayed AE, Hanafi HA, Beavers GM, Zeichner BC. Susceptibility of sand flies to selected insecticides in North Africa and the Middle East. J Am Mosq Control Assoc. 2001;17:23–7. 11345414

[37] Azarm A, Vatandoost H, Koosha M, Ahmad Akhavan A, Mohebali M, Saeidi Z, et al. Susceptibility of *Phlebotomus papatasi* (Diptera: Psychodidae) against DDT and deltamethrin in an endemic focus of zoonotic cutaneous leishmaniasis in Iran. J Arthropod Borne Dis. 2023;17:333–43. 10.18502/jad.v17i4.15296. 38868678 PMC11164618

[38] de Araújo Barbosa V, de Souza CF, Pereira A, Gatherer D, Brazil RP, Bray DP, et al. Insecticide-impregnated netting: a surface treatment for killing *Lutzomyia longipalpis* (Diptera: Psychodidae), the vector of *Leishmania infantum*. Curr Res Parasitol Vector Borne Dis. 2021;1:100044. 10.1016/j.crpvbd.2021.100044. 35005688 PMC8716342

[39] Ortuño M, Muñoz-Hernández C, Risueño J, Jumakanova Z, Farinella A, Vaselek S, et al. Effect of high-volume insecticide spraying on sand fly vectors in household gardens in Spain. Zoonoses Public Health. 2023;70:511–22. 10.1111/zph.13062. 37264760

[40] Santamaría E, Cabrera OL, Pardo RH. Efficacy of factory-treated and dip-it-yourself long-lasting insecticide-treated bednets against cutaneous leishmaniasis vectors in the sub-Andean region of Colombia: results after two years of use. Mem Inst Oswaldo Cruz. 2020;115:e190431. 10.1590/0074-02760190431. 32935748 PMC7491276

[41] Fawaz EY, Zayed AB, Fahmy NT, Villinski JT, Hoel DF, Diclaro JW 2nd. Pyrethroid insecticide resistance mechanisms in the adult *Phlebotomus papatasi* (Diptera: *Psychodidae*). J Med Entomol. 2016;53:620–8. 10.1093/jme/tjv256. 26810731

